# Assessments of Ground Reaction Force and Range of Motion in Terms of Fatigue during the Body Weight Squat

**DOI:** 10.3390/ijerph18084005

**Published:** 2021-04-11

**Authors:** Berkant Erman, Mehmet Zeki Ozkol, Jelena Ivanović, Hakan Arslan, Marko Ćosić, Yasin Yuzbasioglu, Milivoj Dopsaj, Tolga Aksit

**Affiliations:** 1 Department of Sport Health Sciences, Institute of Health Sciences, Ege University, 35050 Izmir, Turkey; berkanterman@hotmail.com (B.E.); egehakanarslan@gmail.com (H.A.); 2 Coaching Education Department, Faculty of Sport Sciences, Ege University, 35050 Izmir, Turkey; yasin.yuzbasioglu@ege.edu.tr (Y.Y.); tolga.aksit@ege.edu.tr (T.A.); 3 Serbian Institute of Sport and Sports Medicine, 72 Kneza Višeslava Street, 11030 Belgrade, Serbia; jelena.ivanovic@rzsport.gov.rs; 4 Theory of Sports Training, Faculty of Sport and Physical Education, University of Belgrade, 11000 Belgrade, Serbia; marko.cosic@fsfv.bg.ac.rs; 5 Faculty of Sport and Physical Education, University of Belgrade, 11000 Belgrade, Serbia; milivoj.dopsaj@gmail.com; 6 Institute of Sport, Tourism and Service South Ural State University, 454080 Chelyabinsk, Russia

**Keywords:** angular kinematic, weight distribution, foot position, performance

## Abstract

The purpose of this study was to analyse in detail body weight squat (BWS)’ fatigue effect on the range of motions (ROM) of the hip, knee, ankle and ground reaction forces (GRF). Twenty male recreational athletes (24.0 ± 3.1 years, 178.85 ± 7.12 cm and 78.7 ± 11.45 kg) participated in this study. BWS were performed on four load cell platforms until the participants failed to continue. Participants performed 73 ± 27 repetitions and the duration to complete of the repetitions was 140.72 ± 62.28 s during the BWS exercise. The forefoot and hindfoot of the feet were on two load cells, thus, there were two under each foot. All of the data collected was divided into three sections for analysis (24 ± 9 repetitions for each). In terms of GRF of the fore feet and hind feet, significant differences and medium to large effect size were found between each section (*p* = 0.006~0.040, ES = 0.693~0.492). No significant differences were found between right and left leg in all sections. Significant differences were found in the ROM of the hip between the sections of first-third (*p* = 0.044, ES = 0.482) and second-third (*p* = 0.034, ES = 0.510), the ROM of the knee first-third (*p* = 0.014, ES = 0.602) and second-third (*p* = 0.005, ES = 0.701) and for the ROM of the ankle first-second (*p* = 0.045, ES = 0.479). As a result, end-of-exercise fatigue caused an increase in the ROM of the hip, knee and ankle. Thus, it is observed that fatigue induced increased ROM, also increases the GRF towards the forefeet.

## 1. Introduction

The squats are type of exercises that have a wide range of usage. Primary usage area of this exercise; strength development and rehabilitation. Because of these wide usages, there are many separately important factors in performing the movement healthy and effective. Such as, strength induced factors; symmetrical/asymmetrical ground reaction forces (GRF) [1,2,3], the strength and strength balance of the leg and core muscles [4,5]. Joint induced factors; hip, ankle and trunk mobilisation, the stabilisation of the hip and knee and ligament health [6]. Stance induced factors, the position of the feet [4,5], head position [7] and angle of the ground [8].

Generally, previous literature has analysed the measurement of leg muscle surface electromyography, angular kinematics and GRF during squat with external loads using one or two force plates [9,10,11,12]. Regarding symmetry/asymmetry of the GRF, observing small functional asymmetry is common in these studies [1]. Considering the functionally of the squat exercise programs—even for movement tests—the first step is usually completed without external load, using body weight only [13,14]. This form of assessment makes it difficult to detect asymmetry in healthy participants [15,16]. These situations show that there are different results as to whether there is asymmetry or not. However, healthy learning technique of the squat exercise with weightless sticks or light external loads is generally the initial step for the beginners. Chiu et al. [17] suggest that plate squat is one of the ideal exercises for teaching the initial steps of proper squat technique and that applying just 2 to 10 kg of additional weight will help generate stability and assist in the reflexive activation of the spinal musculature. This may be particularly important in terms of rehabilitative usage of body weight squat (BWS) exercise.

The findings of previous literature show that fatigue is one of the main significant factors affecting BWSs during exercise [1,18,19,20] as any other exercises. Due to the effects of fatigue, it is expected that, if the velocity of a movement is slow, there will be a more symmetrical distribution between the dominant leg and the nondominant leg [18,21].

Poor BWS performance may change GRF and angular stability due to fatigue and this unstable condition may increase the load on the ligaments [18]. On the other hand, external loads with using barbells are require greater torque generation during squat exercises [21]. It may be concluded that the risk of injury may increase in the long term, with varying stability and torque.

In the present study, how the range of motions (ROM) and GRF changes in the state of fatigue, whether there is a relationship between them, and how the constant tempo affects exercise has been investigated in detail. Therefore, by considering these changes, practitioners may perform the BWS exercise more efficiently. It is hypothesised that (1) functional asymmetry would be observed and would continue during the exercise, (2) when fatigue occurred, body weight distribution would shift towards the forefeet, (3) the ROM of the joints (hip, knee and ankle) would decrease when fatigued.

## 2. Materials and Methods

### 2.1. Participants

The present investigation was designed as a cross-sectional, repeated-measures, within subject study. The sample size was calculated using GPower 3.1.9.4 [power size (1-β) = 0.80, the effect size (f) = 0.25, type-1 error (α) = 0.05, number of groups = 1, number of measurements = 3, cor. among rep. measure = 0.5, correction ε = 1]. According to the results of this analysis, sample size was 28. However, twenty recreationally male athletes (Ages—24.0 ± 3.12 years, BH—178.85 ± 7.12 cm and BM—78.7 ± 11.45 kg) volunteered and gave their informed consent to take part in the study. Therefore, the power of the study has dropped to 0.67. Participants were included in the study if they (a) had already practiced and continued squat exercises in a strength training program, (b) participants must have right dominant leg (c) had no chronic limb discomfort or limitations that had a negative effect on exercise, such as pes planus, pes cavus, genu valgum, or genu varum. This study was carried out in a laboratory in two sessions at 72-h intervals. It has been proven by scientific studies that the exercises are affected by time-of-day. Changes in testosterone, cortisol and neuromuscular adaptation have been observed to affect training efficiency [22]. For this reason, it is important at what hour the training is performed. If the competition time is not known, it is recommended to apply resistance exercises in the morning [23]. Therefore, all participants performed the sessions in the morning between 9:00–12:00. The duration to complete of the first session was approximately 45 min and 30 min in the second session for each participant. The aim of the first session was subject’s familiarisation with measurement procedures and BWS protocols, as an exclusion criterion the participants who could not adapt to the metronome were excluded from the study and in the second session, the participants performed the BWS testing protocol. GRF and ROM data was obtained via four platform type load cells and video recording. The experimental procedures undertaken were approved by Ege University’s Medical Research Ethics Committee (19–5.1T/45) and are in agreement with the principles of the Declaration of Helsinki [24]. The participants were briefed on the study procedures and informed consent was obtained before they took part in the study.

### 2.2. Data Collection and Recording Procedures

Each participant performed one set of BWS exercise until they failed to keep up the metronome because of fatigue. The BWSs were performed using a tempo standardised to a two-count cadence at 45 beats per minute (bpm). The cadence was 1:0:1:0, which signifies a one-count eccentric motion followed immediately by a one-count concentric motion, without any count for rest before beginning the next repetition. In the first laboratory session, retroreflective markers were positioned for the acromion (shoulder), iliac spine (hip), epicondylus lateralis (knee), malleolus lateralis (ankle) and caput metatarsalis (toe) points before the BWS exercise. After the participants had done a 10-min individual warm up, with 5 min of passive rest, they stepped on each load cell with the left and right foot. Before first testing session, each load cell was calibrated according to standard participants body mass procedures. The forefoot and hindfoot parts of the feet were on two load cells, thus the midfoot section of each foot was located on the border of the load cells. There were two under each foot. Next, the metronome was started (45 bpm) and the BWS exercise was performed until the participant could adapt to the metronome.

In the second laboratory session, after the participants stepped on four load cells at once, each load cell was calibrated according to their body weight distribution. In order to ensure that each load cell was placed symmetrically next to each other, load cells feet was carefully adjusted using a spirit level. To obtain the GRF data, four platform type loadcell (SPS Platform 60 × 60 cm, CAS Electronic Industry and Trade Inc., Korea) were connected to a data acquisition system (BIOPAC MP150, Biopac Systems, Goleta, CA, USA) and the system was reset to zero (load cell signal range; 0–0.15 kg) before the participant stood on the load cells. The data was recorded continuously at 2000 Hz with using AcqKnowledge (4.4.2) software. The BWS exercise was captured by a Basler Ace acA1300-200uc camera (Basler AG, Ahrensburg, Germany) with a frequency of 100 Hz. The camera was placed on the lateral (the right-dominant-foot) side of the participants. The retroreflective markers were placed at the same reference points as in the first laboratory session. A digital metronome was used to standardise the exercise tempo (45 bpm, 1:0:1:0). After the participants had warmed up, they stepped on each load cell (two load cells under each foot), and with their feet approximately opened hip width. They then performed the BWS exercise looking straight ahead at a fixed point that had been placed on the wall (a black dot). The participants raised their arms at the descent phase and released their arms to avoid the fatigue of the arms during the ascent phase. The depth of the squat was self-selected. No feedback or warnings were given to the participants during the BWS exercise in order to observe the possible ROM distortions and asymmetrical weight distributions (the GRF on load cells, right and left forefoot-hindfoot) related to the fatigue. The exercise was performed until participants failed to keep up the metronome because of fatigue. The GRF and ROM data series were divided into three equal (BWS repetitions) sections for observing the effects of fatigue on exercise. The sections of GRF data were then filtered using a low pass infinite impulse response (IIR) filter at 50 Hz and the all-signal wave got averaged via AcqKnowledge (4.4.2) software. The sections of ROM data were analysed with a Kinovea (0.8.27) motion analyse software program (downloaded at:www.kinovea.org (accessed on 6 September 2019). In the BWS exercise, the last point of upward position was taken as extension angle and the last point of downward position was taken as flexion angle. The differences between these angles allowed us to obtain the ROM of hip, knee and ankle.

### 2.3. Statistics and Data Analysis

The recorded GRF and ROM data series divided into three sections for analysis by averaging the values of each tercile section (1st, 2nd, 3rd). For each section of GRF and ROM, data were presented as the mean and standard deviation. The Shapiro–Wilks test was used to check for normality and normality assumptions was met. To assess differences between the tercile sections, one-way repeated-measures analysis of variance (ANOVA) was used. Greenhouse–Geisser adjustments of the *p*-values were reported, since sphericity assumption was violated (*p* < 0.05). Least significant difference (LSD) was used for multiple comparisons. Paired *t*-test was performed for matched pairs of GRF within same section. Cohen’s *d* was used as an effect size and categorised as no effect (0–0.2), small effect (0.2–0.5), medium effect (0.5–0.8), and large effect (>0.8) [25]. Test-retest reliability for the GRF and ROM between the first, second and third sections was analysed by ICC. The ICC are reported with 95% of confidence interval (95% CI) and categorised as poor (<0.5), moderate (0.5–0.75), good (0.75–0.9) and excellent (>0.9) [26]. Standard error of measurement (SEM) values were calculated for the tercile sections. Pearson correlation coefficients (r) were used to determine the relationship between the ROMs and GRFs. Data analysis was performed using SPSS software (IBM SPSS Statistics for Windows, Version 25.0. Armonk, NY: IBM Corp). The statistical significance level was set at *p* ≤ 0.05.

## 3. Results

The one-way repeated ANOVA results showed that there was significant difference in the forefoot GRF of the right leg. Additionally, significant difference was found in the fore, hind and total (fore + hind) GRF variables in the left leg, also in the forefoot (right + left) and hindfoot (right + left). As a result of the angular evaluation, except for the extension angle, there was significant difference in flexion angle and ROM of the knee joint. In the hip and ankle joints, there was only a significant difference in the flexion angle. There was not observed any significant difference in the extension angle and ROM and all multiple comparisons of ANOVA results were also given in Table 1.

**Figure 1:**
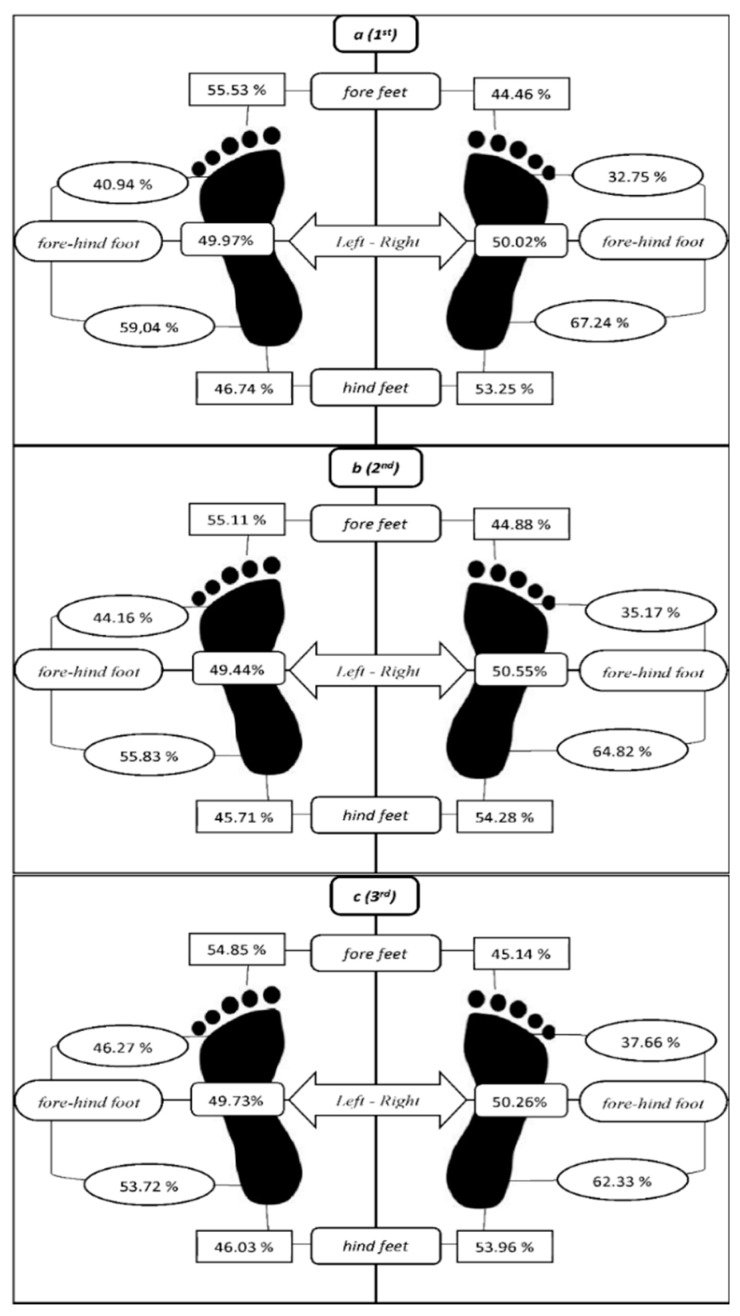
Weight distribution percentages of all sections (**a**) First section, (**b**) Second section, (**c**) Third section.

In the evaluation of the GRF values via paired *t*-test, there were significant differences in all matched-pairs (0.001 < *p* < 0.0024, 0.024 < ES < 0.34) except “leg total (right vs. left)” in the first section. In the second section, there were significant differences in all matched-pairs (0.001 < *p* <0.005, 0.034 < ES < 1.4) except “total (right vs. left)” and “left foot (fore vs. hind)”. In the third section, there were significant differences in all matched-pairs (0.002 < *p* < 0.033, 0.034 < ES < 1.15) except “total (right vs. left)” and “left foot (fore vs. hind)” (Table 2). Distributions of the GRFs are shown in Figure 1 for each section.

The test-retest reliability for GRFs and ROMs also shown that there was good to excellent reliability between the tercile sections (0.79 < ICC < 0.98) (Table 3).

Bivariate correlations between ankle, knee, hip ROM and right leg GRF variables are presented in Table 4. Significant negative relationship was found in three bivariate correlations (−0.623 < *r* < −0.449, *p* < 0.05). There were no other significant correlations between the ROM and GRF.

**Figure 2:**
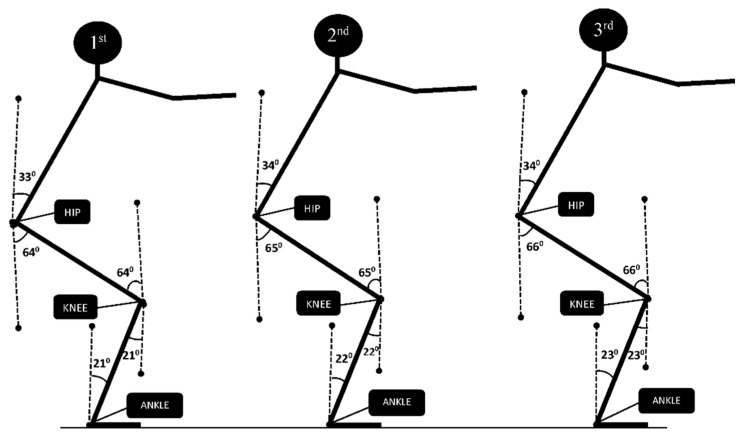
The averages of the ROM of 1st, 2nd, 3rd sections.

The ROM of the hip, knee and ankle were in the first section; 97° (33° + 64°), 85° (21° + 64°) and 21°, in the second section, they were 99° (34° + 65°), 87° (22° + 65°) and 22° and in the third section, they were 100° (34° + 66°), 89° (23° + 66°) and 23° (Figure 2).

## 4. Discussion

The current study examined how fatigue effects the ground reaction forces and range of motions during the body weight squat exercise. Thus, the squat performance was divided into three equal sections in order to evaluate fatigue. Considering the fatigue, changes were observed in the ground reaction forces and the range of motions. The ground reaction forces shifted towards the forefeet and range of motions increased with fatigue. It was observed that when the hip range of motion increased, the right foot total ground reaction force decreased. In addition, the strong reliability was observed between the sections of BWS exercise.

### 4.1. First Section of BWS Exercise

There was no significant difference between the GRFs of the right and left foot. This finding does not support first hypothesis as there was no functional asymmetry in the healthy participants studied. While some studies have reported that functional asymmetry is quite normal [1]. The percentages of body weight distribution support this outcome (left: 49.97%–right: 50.02%). However, there was a significant difference between fore and hind parts of the feet. The general standing posture may be explained by the concentration of the body weight distribution in the hind part of the feet. Considering that the participants carefully placed the fore and hind parts of their feet when performing the exercise on the load cell platforms, it is possible to say that the participants were supported by the hind part of their feet during the first section.

The extensions, flexions and ROM of the hip, knee and ankle during each section were also examined. It may be seen that the knees were slightly bent during the extension phase. Therefore, it may be said that the exercise load was distributed well for muscles rather than joints. For this study, the depth of the squat movement was not restricted by the researchers. During the descending phase of the knee, flexion was just 3° below 90°. This value shows that they tended to perform half squats (70° to 100°) [4] without verbal encouragement or guidance. However, it has been shown that more power output can be produced by a deep squat [27] and that the gluteus maximus muscle is more active when it is below 90° [28]. In addition, a larger feet stance increases tight muscle activations such as, rectus femoris, vastus lateralis and adductor longus [29,30]. On the contrary, knee flexion of below 90° with symmetry may increase stiffness or restricted joint mobility/stability [30]. Thus, it may be said that guidance must be given for BWS, but care has to be taken for the proper form of squat.

### 4.2. Second Section of BWS Exercise

When examining the second section, the difference regarding the fore and hind parts of the left foot between the first and second sections of GRF are gradually closing up. However, the participants continued exercising by maintaining the difference between the fore and hind parts of the right foot in the first and second section. Considering that all of the participants in this study had been right leg dominant, this may be interpreted as an indication that the support leg (left fore) gradually transfers more force from hind to fore on the ground when fatigue occurs. Considering this transfer, the second section may be said that “the beginning of the increasing fatigue section”. Regarding the GRF, weight distribution transferred to the right and left forefeet from the hind feet. Hindfeet contribution decreased from 63.1% to 60.3% in the second section. Dioniso et al. [9] research on healthy participants found that the deceleration phase of a squat is characterised by the centre of pressure (COP) displacement to the tip of the toe. This study, which uses load cell instrumentation instead of the force plate, supports this outcome and it could be said that the deceleration phase of squat may be one of the reasons for the forward transmission of the body weight distribution with fatigue.

The average hip extension in the second section remained the same as in the first section, while the flexion value increased. It may be concluded that only the hip may not be held responsible for the shifting of the weight distribution, which is contrary to some studies [27,31,32]. The ROM of the ankle significantly increased by 10% compared to the first section.

It could be said that the GRF transfer was increasing more forward due to these increased ROMs. These findings support this study’s second hypothesis while do not support its third hypothesis.

### 4.3. Third Section of BWS Exercise and Comparison of All Sections

There was no difference between the GRF of the participants’ dominant leg (right) and support leg (left) in third section and also in all sections. Flanagan et al. [1] reported that during a squat with using external load, the GRF in the support leg is greater. Conversely, this study shows that there is no such tendency (leg dominance), and the support leg GRFs were almost same but lower than the dominant leg in all sections.

No information could be found in the literature on the GRF of the fore and hind parts of the feet during the whole exercise. In this study, especially during the third section, the difference in the distribution of the GRF between the fore and hind part of the left foot (the support leg) decreased compared to the previous sections and the differences between the fore and hind part of the right foot (the dominant leg) show an irregularly distributed GRF during all sections. Interestingly, although the fore and hind part of the foot is not expected to carry the same load, the fore-hind disparity of the dominant leg did not close during the progressive sections of the BWS exercise. This distribution could be considered as an indication that the support leg (the left leg) is more active than the dominant leg (the right leg) towards the end of the exercise to compensate for the distribution of body weight. However, this highlights one of this study’s limitations, which is that electromyogram (EMG) would show muscular activity and this could be named as the reason why. Previous studies report that there is no difference when fatigue occurs [10,19] and this study supports of these studies findings on the fatigue effect.

During the exercise, the body weight was transferred to the forefeet during each section and increased to 5.1% in the last section compared to the first section when there was symmetry between left and right leg of the participants. This is thought to be caused by changes in the ROM. The hip extension angle in the third section was found to be constant at 174° as it was in the first two sections. The flexion of the hip decreased to 73° and the tendency of bringing the hip to the front increased and the ROM enhanced. The ROM of the knee in the third section increased by 4° compared to the first. In fact, the increase in this ROM suggests that adaptation to the metronome gradually deteriorated, especially in last repetitions, participants released themselves to the downward phase more rapidly due to the fatigue. Although performing squats without locking the knees over a long period of time, the slightly increased in the velocity of the movement in acceleration phase due to the catch up the metronome might cause injury in the knees. This situation could increase the load on the knee by 28% as well [18].

A study in the literature used 92 bpm for a fast tempo and 54 bpm for a slow tempo and an external load was used [33]. The metronome tempo used in this study is thought to be as effective as a comfortable squat tempo with body weight (45 bpm represents approximately 1.3 s for each phase). There is a gap in the literature for observing resistance exercises (external loaded or unloaded) with using a metronome. A standardised pace will undoubtedly offer an opportunity to better compare the literature with each other.

Another explanation for the increase in ROM may be the fact that the body weight was transferred forward and only trunk flexion was not responsible for this. The increased ROM of the hip and knee observed in the last section shows that the load on the hind feet was gradually distributed forward from the beginning of the exercise. This study supports the notion that increased ROM affects the GRF and this shifts to the forefoot [9].

Considering this study, captured only one lateral side in 2D to assess the ROM (the right-dominant-foot), for that reason, the GRF and ROM of the right side would provide a better evaluation for correlation analysis. Significant negative relationships were found between the ROM of the hip and the GRF of the right foot, ROMs explain 30% of variation in GRF. Additionally, in the second section that increasing of the ROM of the hip, knee and ankle resulting with gradual deterioration of stability due to the fatigue. These increases are thought to be due to the continuation of the downward (acceleration) phase of the exercise by the participants as they are trying to catch up to the metronome tempo. There is also a significant negative relationship between the ROM of the knee, hip and the GRF of the right foot in the third section, ROMs explain 20% and 38% of variation in GRF, respectively. Thus, it may be noticed that one of the primary factors was the ROM of knee and hip when shifting weight to the forefeet due to the constant speed and fatigue. In addition, the hip showed better correlations than the knee joint.

## 5. Conclusions

According to the findings of this study, it is the ROM of the hip and knee that primarily has an effect on GRF shifting. Because of this, coaches should be aware of the need to observe whole performance patterns. Thus, coaches should not push individuals too hard, especially during the last phase of a workout (the manifested fatigued phase of squat exercise) when exercising at a constant speed, as this would increase ROM and may cause injury in the long term. For practical considerations, movement screen is may made at the beginning of the exercise, and the squat performed with light loads or body weight will increase the benefit of the practitioners. They may be advised to strengthen their core muscles in order to have a more controlled ROM in case of fatigue. Thus, they can be protected from long-term undesirable consequences (i.e., injury). For experimental studies, this study may be improved by further researches through more analysis, placing weight shifts on the force plates for the weight distribution of the body for each foot, 3D analysis of ROM, also using EMG and exploring the different constant tempo effects of using a metronome during whole BWS exercise.

## Figures and Tables

**Table 1:** The one-way repeated ANOVA results and multiple comparisons.

	First Section	Second Section	Third Section			First vs. Second Section			First vs. Third Section			Second vs. Third Section		
	M ± SD	M ± SD	M ± SD	Repeated ANOVA *p*	$n_{p}^{2}$	diff.	Pairwise *p*	ES	diff.	Pairwise *p*	ES	diff.	Pairwise *p*	ES
R_Forefoot	11.98 ± 5.41	12.98 ± 5.40	13.70 ± 5.76	0.038 *	0.214	−1.00	0.091	0.39	−1.72	0.035 *	0.5	−0.72	0.062	0.43
R_Hindfoot	24.59 ± 6.99	23.92 ± 6.36	22.66 ± 6.52	0.066	0.151	0.67	0.264	0.25	1.93	0.063	0.44	1.25	0.062	0.44
R_Foot total	36.57 ± 5.36	36.90 ± 5.21	36.37 ± 5.15	0.429	0.35	−0.32	0.052	0.45	0.20	0.718	0.08	0.53	0.334	0.22
L_Forefoot	14.96 ± 7.14	15.94 ± 7.05	16.65 ± 7.6	0.008 *	0.258	−0.98	0.040 *	0.49	−1.69	0.009 *	0.65	−0.70	0.054	0.46
L_Hindfoot	21.58 ± 6.81	20.14 ± 6.33	19.33 ± 6.51	0.001 *	0.377	1.43	0.003 *	0.34	2.24	0.001 *	0.49	0.81	0.049 *	0.46
L_Foot total	36.54 ± 7.03	36.09 ± 7.16	35.98 ± 7.02	0.008 *	0.233	0.44	0.011 *	0.05	0.55	0.011 *	0.63	0.10	0.508	0.02
Forefoot_(R+L)	26.94 ± 12.21	28.92 ± 12.05	30.36 ± 12.95	0.01 *	0.259	−1.98	0.040 *	0.11	−3.42	0.010 *	0.23	−1.43	0.039 *	0.11
Hindfoot_(R+L)	46.17 ± 12.99	44.07 ± 12.02	41.99 ± 12.37	0.005 *	0.289	2.10	0.033 *	0.11	4.18	0.006 *	0.24	2.07	0.027 *	0.17
Knee_Ext	172.30 ± 3.74	171.50 ± 3.42	172.70 ± 4.00	0.194	0.085	0.80	0.176	0.23	−0.40	0.631	0.0	−1.20	0.033 *	0.2
Knee_Flex	87.25 ± 18.75	84.10 ± 19.19	83.15 ± 19.49	0.015 *	0.253	3.15	0.010 *	0.11	4.10	0.019 *	0.15	0.95	0.200	0.03
Knee_ROM	85.05 ± 18.32	87.40 ± 18.46	89.55 ± 18.1	0.016 *	0.248	−2.35	0.077	0.41	−4.50	0.014 *	0.6	−2.15	0.005 *	0.69
Hip_Ext	174.80 ± 5.44	174.35 ± 5.06	174.50 ± 4.23	0.78	0.008	0.45	0.469	0.0	0.30	0.777	0.0	−0.15	0.842	0.0
Hip _Flex	77.30 ± 14.47	75.35 ± 15.74	73.55 ± 15.58	0.011 *	0.234	1.95	0.051	0.09	3.75	0.012 *	0.19	1.80	0.079	0.09
Hip _ROM	97.50 ± 13.99	99.00 ± 15.57	100.95 ± 16.22	0.054	0.161	−1.50	0.256	0.26	−3.45	0.044 *	0.48	−1.95	0.034 *	0.51
Ankle_Ext	90.15 ± 4.27	89.30 ± 3.62	89.20 ± 4.23	0.256	0.068	0.85	0.193	0.2	0.95	0.266	0.17	0.10	0.748	0.0
Ankle_Flex	68.65 ± 8.45	66.60 ± 8.64	66.30 ± 8.66	0.029 *	0.206	2.05	0.012 *	0.17	2.35	0.042 *	0.17	0.30	0.587	0.0
Ankle_ROM	21.50 ± 6.82	22.70 ± 6.65	22.90 ± 6.71	0.063	0.152	−1.20	0.045 *	0.47	−1.40	0.072	0.15	−0.20	0.618	0.28

* p ≤ 0.05; M ± SD: Mean ± Standard Deviation; $n_{p}^{2}:\;\mathrm{Partial}\;\mathrm{eta}\;\mathrm{squared};\;\mathrm{diff}:\;\mathrm{Mean}\;\mathrm{difference};\;$ ES: Effect size; Foot total: Forefoot + Hindfoot; R: Right; L: Left; Ext: Extension; Flex: Flexion; ROM: Range of motion.

**Table 2:** Paired *t*-test results.

	First Section			Second Section			Third Section		
	MD	p	ES	MD	p	ES	MD	p	ES
Right (Fore vs. Hind) Foot	−12.61	0.001 *	1.66	−10.94	0.001 *	1.4	−8.95	0.002 *	1.15
Left (Fore vs. Hind) Foot	−6.61	0.024 *	0.75	−4.20	0.114	0.54	−2.67	0.343	0.32
Fore (Right vs. Left) Feet	−2.98	0.001 *	0.34	−2.96	0.001 *	0.34	−2.94	0.003 *	0.34
Hind (Right vs. Left) Feet	3.01	0.010 *	0.35	3.77	0.001 *	0.34	3.33	0.002 *	0.35
Total (Right vs. Left)	0.034	0.974	0.0	0.81	0.484	0.0	0.38	0.715	0.11
Total (Fore vs. Hind)	−19.23	0.001 *	1.17	−15.14	0.005 *	0.94	−11.63	0.033 *	0.64

* *p* ≤ 0.05; MD: Mean Difference; ES: Effect size.

**Table 3:** The test-retest reliability between sections.

	First–Second Section			First–Third Section			Second–Third Section		
	SEM	ICC (*p*)	ICC 95% CI	SEM	ICC (*p*)	ICC 95% CI	SEM	ICC (*p*)	ICC 95% CI
			Lower–Upper			Lower–Upper			Lower–Upper
R_Forefoot	0.56	0.89 (0.01)	0.74–0.95	0.75	0.81(0.01)	0.59–0.92	0.36	0.95 (0.01)	0.89–0.98
R_Hindfoot	0.58	0.92 (0.01)	0.81–0.96	0.97	0.79 (0.01)	0.54–0.91	0.63	0.9 (0.01)	0.77–0.96
L_Forefoot	0.44	0.96 (0.01)	0.9–0.98	0.58	0.93 (0.01)	0.85–0.97	0.34	0.97 (0.01)	0.94–99
L_Hindfoot	0.42	0.95 (0.01)	0.89–0.98	0.58	0.92 (0.01)	0.81–0.96	0.38	0.96 (0.01)	0.91–0.98
Knee_ROM	1.25	0.95 (0.01)	0.88–0.98	1.67	0.91 (0.01)	0.8–0.96	0.68	0.98 (0.01)	0.96–0.99
Hip _ROM	1.28	0.92 (0.01)	0.82–0.097	1.6	0.88 (0.01)	0.74–0.95	0.85	0.97 (0.01)	0.92–0.98
Ankle_ROM	0.56	0.91 (0.01)	0.83–0.97	0.73	0.88 (0.01)	0.72–0.95	0.39	0.96 (0.01)	0.91–0.98

**Table 4:** Bivariate correlations between ROM and GRF at three sections.

	Sections		RFF GRF	RHF GRF	RTOT GRF
KNEE ROM	First	*r*	0.086	−0.229	−0.212
		*p*	0.718	0.331	0.369
	Second	*r*	0.001	−0.339	−0.413
		*p*	0.998	0.144	0.070
	Third	*r*	−0.045	−0.314	−0.449
		*p*	0.849	0.177	0.047 *
HIP ROM	First	*r*	−0.129	−0.174	−0.357
		*p*	0.588	0.462	0.122
	Second	*r*	−0.304	−0.199	−0.557
		*p*	0.193	0.401	0.011 *
	Third	*r*	−0.339	−0.192	−0.623
		*p*	0.144	0.416	0.003 *
ANKLE ROM	First	*r*	0.155	−0.186	−0.086
		*p*	0.515	0.433	0.718
	Second	*r*	0.110	−0.196	−0.125
		*p*	0.643	0.408	0.600
	Third	*r*	0.106	−0.163	−0.088
		*p*	0.656	0.492	0.713

* p ≤ 0.05; RFF: Right Forefoot; RHF: Right Hindfoot; RTOT: Right Foot Total (Fore + Hind).

## Data Availability

The data presented in this study are available on request from the corresponding author.
